# Targeting MDM2 homodimer and heterodimer disruption with DRx-098D in *TP53* wild-type and mutant cancer cells

**DOI:** 10.1016/j.omton.2025.201029

**Published:** 2025-08-06

**Authors:** Sean F. Cooke, Thomas A. Wright, Gillian Lappin, Elka Kyurkchieva, Yuan Yan Sin, Jiayue Ling, Alina Zorn, Bria O’Gorman, William Banyard, Chih-Jung Chang, Helen Wheadon, Danny T. Huang, George S. Baillie, Connor M. Blair

**Affiliations:** 1 College of Medical, Veterinary and Life Sciences, University of Glasgow, G12 8QQ Glasgow, UK; 2 Paul O’Gorman Leukaemia Research Centre, University of Glasgow, G11 0YN Glasgow, UK; 3 Cancer Research UK – Scotland Institute, G61 1BD Glasgow, UK

**Keywords:** MT: Regular Issue, MDM2, MDMX, dimerization, disruptor peptide, TP53 mutant cancer

## Abstract

Novel pharmacological strategies capable of inhibiting pro-oncogenic MDM2 beyond its p53-dependent functions represent increasingly attractive therapeutic strategies to treat solid and hematological cancers that are dependent upon MDM2/MDMX, regardless of *TP53* mutational status. Utilizing a novel first-in-class cell-penetrating peptide disruptor of MDM2 homo- and heterodimerization (DRx-098D), we demonstrate the anti-proliferative potential of blocking MDM2 dimerization against a panel of human cancer cell lines that are *TP53* wild type, mutant, or null. DRx-098D elicits its anti-cancer activity via a differentiated mechanism vs. idasanutlin (a phase 3 clinical candidate MDM2-p53 small-molecule inhibitor), inducing significantly superior growth inhibition against *TP53* null HCT116 cells. Our preliminary data highlight, for the first time, the potential therapeutic utility of exploiting both MDM2 homo- and heterodimerization in *TP53* wild-type and mutant cancers with an MDM2-derived disruptor peptide.

## Graphical abstract

**Figure:**
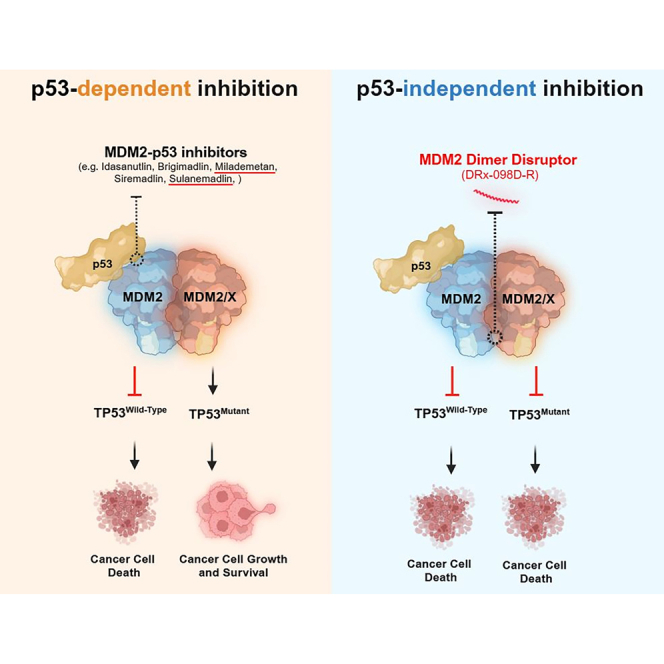

## Teaser

New approaches to neutralizing MDM2 in cancer are urgently needed. This is particularly true for cancers that harbor a mutation in the TP53 gene, rendering existing clinical-stage MDM2-targeted therapies redundant. Blair and colleagues have discovered a novel strategy to inhibit MDM2 across a broad range of cancers, including those with a TP53 mutation.

## Introduction

Growing recognition of the pro-oncogenic influence, which MDM2 and MDMX have in promoting cancer survival and metastasis, independent of p53, exemplifies the urgent unmet need for novel therapeutic approaches to exploiting MDM2 and MDMX activity.^1^^,^^2^^,^^3^ Current clinical candidate MDM2-targeting therapeutics (idasanutlin, milademetan, siremadlin, brigimadlin, and sulanemadlin) specifically block MDM2/MDMX’s ability to negatively regulate p53 transcriptional activity and promote its degradation via the ubiquitin proteosome system.^4^^,^^5^ Though this approach has proven successful in *TP53* wild-type (WT) cancer, it does not translate to cancers harboring a *TP53* mutation (MT).^5^ In this context, MDM2/MDMX’s role in suppressing p53 diminishes. Instead, MDM2/MDMX continue to drive tumorigenesis through a myriad of p53-independent mechanisms, an area of research that remains within its infancy.^1^^,^^2^^,^^3^^,^^4^^,^^5^

MDM2 forms homodimers (MDM2:MDM2) and heterodimers (MDM2:MDMX) via its C-terminal RING domain, essential for its enzymatic E3 ligase activity.^6^^,^^7^^,^^8^^,^^9^ Consequently, pharmacologically targeting the de-stabilization/disruption of C-terminal RING domain dimerization is considered an attractive and underexploited approach to inhibiting MDM2/MDMX, offering a potentially viable therapeutic strategy against MDM2/MDMX-dependent cancers, irrespective of *TP53* mutational status.^5^^,^^10^

DRx-098D-R is a novel investigative cell-penetrating MDM2-derived peptide designed to disrupt MDM2:MDM2 and MDM2:MDMX at the dimerization interface. This proof-of-concept study outlines DRx-098D-R’s ability to (1) directly bind MDM2 and MDMX, (2) disrupt intracellular MDM2 homo- and heterodimerization, (3) inhibit MDM2 E3 ligase activity, and (4) promote cancer cell death across a broad spectrum of *TP53* WT and MT human cancer cell models. These data re-enforce the therapeutic value of targeting the disruption of pathological protein complexes in disease and precision medicine.^11^^,^^12^^,^^13^

## Results

### DRx-098D-R directly binds MDM2 and MDMX RING-C-terminal truncate proteins

**Figure 1:**
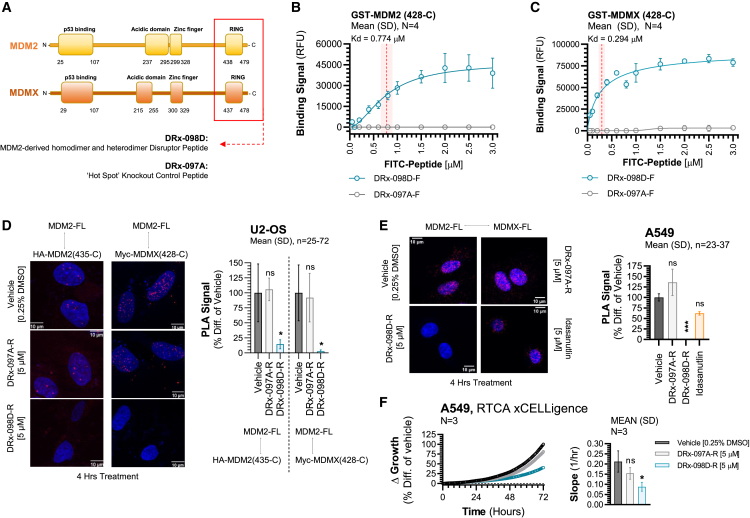
Validation of DRx-098D on-target binding and PPI disruption (A) MDM2/MDMX structural domains. Red box highlights the MDM2:MDMX RING-C-terminal domain exploited by DRx-098D. Target engagement of (B) GST-MDM2 (428-C) and (C) GST-MDMX (428-C) with FITC-labeled DRx-098D and DRx-097A. (D) Proximity ligation assay (PLA) of full-length (FL) MDM2 and HA-MDM2(435-C) or Myc-MDMX(428-C) in U2-OS, treated with vehicle, DRx-098D-R, or DRx-097A-R. (E) PLA of endogenous FL-MDM2 and MDMX in A549, treated with vehicle, DRx-098D-R, DRx-097A-R, or idasanutlin. (F) A549 real-time cell growth analysis (xCELLigence) following 72 h treatment with vehicle, DRx-098D-R, and DRx-097A-R. ns, not significant; ∗*p* < 0.05; ∗∗*p* < 0.01; ∗∗∗*p* < 0.001.

MDM2 forms homodimers and heterodimers with MDMX through its RING-C-terminal domain, and a disruptor peptide, DRx-098D, was designed to target this region (Figure 1A). To confirm target engagement, glutathione S-transferase (GST) fusion MDM2(428-C) or MDMX(428-C) protein was co-incubated with increasing concentrations (0.05–3 μM) of N-FITC-labeled DRx-098D (DRx-098D-F) or DRx-097A (DRx-097A-F, negative control “knockout” peptide) (Figures 1B and 1C). Unlike DRx-097A-F, DRx-098D-F directly bound to immobilized MDM2 (K_d_ = 0.774 μM) and MDMX (K_d_ = 0.294 μM).

### DRx-098D-R disrupts MDM2 homodimer and heterodimer formation

To achieve cell permeability, DRx-098D was conjugated to a short-sequence cationic (arginine rich) peptide (DRx-098D-R) and assessed for its ability to block endogenous and exogenous MDM2 homo-/heterodimers. In U2-OS cells (*TP53* WT) overexpressing HA-MDM2(435-C), DRx-098D-R significantly downregulated homodimer formation with full-length (FL) endogenous MDM2 (Figure 1D). This was also observed in U2-OS cells overexpressing Myc-MDMX(428-C), where heterodimerization with FL-MDM2 was significantly inhibited. DRx-097A-R did not affect MDM2 dimer formation. In A549 cells (*TP53* WT), DRx-098D-R significantly attenuated endogenous MDM2 heterodimerization (Figure 1E). Again, this was not observed with DRx-097A-R. Idasanutlin, an MDM2-p53 inhibitor, also did not affect MDM2 heterodimerization, further differentiating DRx-098D-R’s MDM2 inhibitory mechanism. Notably, disrupting MDM2 heterodimerization with DRx-098D-R (but not DRx-097A-R) significantly inhibited relative A549 cell growth (Figure 1F).

### MDM2 dimer disruption promotes p53 stabilization and upregulates apoptotic pathways

**Figure 2:**
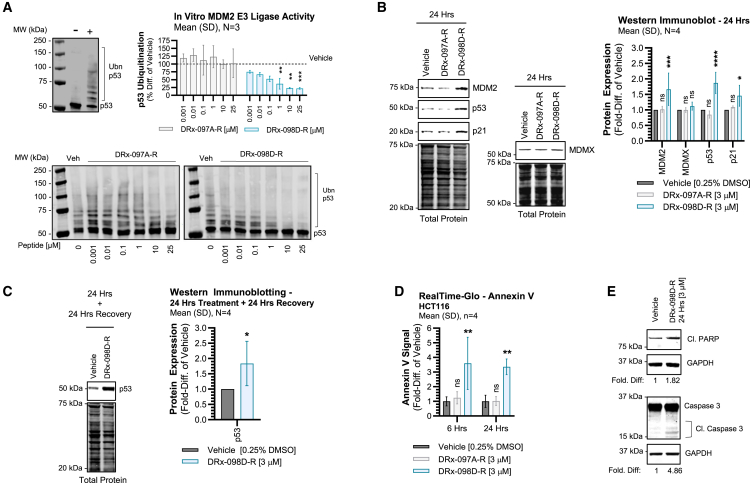
DRx-098D effect on p53 regulation and apoptosis (A) *In vitro* MDM2 E3 ligase activity assay assessing p53 ubiquitination via western immunoblotting analysis. Increasing concentrations of DRx-098D-R or DRx-097A-R were added to the reaction, and relative MDM2-mediated p53 ubiquitination was measured as a percentage difference of vehicle. Negative (−) indicates no Mg^2+−^ATP was in reaction mixture (negative control). Positive (+) indicates all reagents, including Mg^2+−^ATP, were in reaction mixture (positive control) (B) MDM2, MDMX, p53, and p21 protein expression in HCT116^*TP53*^^WT^ following 24 h treatment with vehicle, DRx-097A-R, and DRx-098D-R or (C) 24 h treatment +24 h wash-out with vehicle and DRx-098D-R (expression normalized to total protein). (D) Annexin V levels in HCT116^TP53 WT^ measured after 6 and 24 h treatment with vehicle, DRx-097A-R, and DRx-098D-R. (E) Cleaved PARP and cleaved caspase-3 protein levels in HCT116^*TP53*^^WT^ after 24 h treatment with vehicle and DRx-098D-R. ns, not significant; ∗*p* < 0.05; ∗∗*p* < 0.01; ∗∗∗*p* < 0.001; ∗∗∗∗*p* < 0.0001*.*

To determine whether disrupting MDM2 dimerization would hinder its E3 ligase activity, DRx-098D-R was assessed in a cell-free *in vitro* MDM2-p53 ubiquitination assay (Figure 2A). DRx-098D-R, but not DRx-097A-R, induced a dose-dependent inhibition of p53 ubiquitination. Thus, DRx-098D-R can inhibit MDM2’s E3 ligase activity.

In *TP53* WT cancer, current MDM2/MDMX-p53 inhibitors promote cellular apoptosis as a consequence of upregulated p53 tumor suppressor expression/activity. DRx-098D-R-mediated MDM2 dimer disruption appears consistent with this mechanism, significantly upregulating MDM2, p53, and p21 protein expression (not MDMX) (Figure 2B). In addition, DRx-098D-R-induced elevated p53 levels were sustained following a 24 h treatment recovery period (Figure 2C). DRx-098D-R also promoted increased expression of pro-apoptotic markers Annexin V, cleaved PARP, and cleaved caspase-3 (Figures 2D and 2E).

### DRx-098D-R is anti-proliferative in *TP53* WT and MT/null cell lines

**Figure 3:**
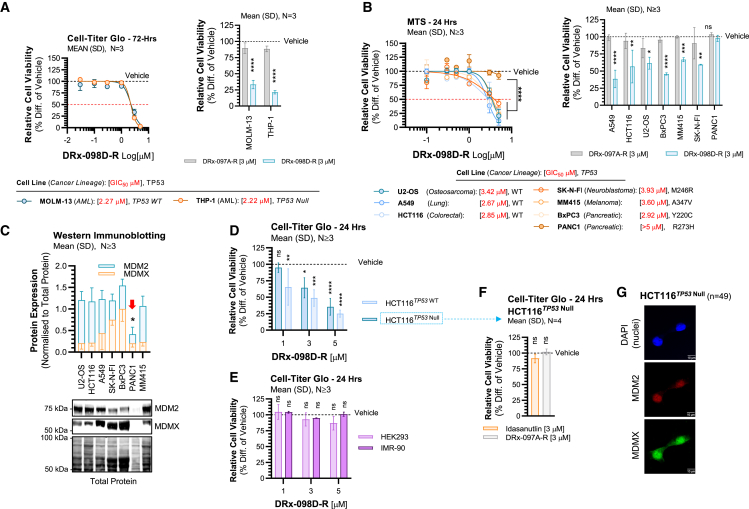
DRx-098D vs. *TP53* WT and MT/null human cancer cell lines (A) Cell viability of human acute myeloid leukemia (AML) cell lines treated with vehicle, DRx-098D-R, or DRx-097A-R for 72 h. (B) Cell viability of a panel of *TP53* WT and MT human cancer cell lines, treated with vehicle, DRx-098D-R, or DRx-097A-R for 24 h. Relative growth (G) IC_50_ (red) is given below. (C) Combined MDM2 and MDMX protein expression. (D) Cell viability of cancerous HCT116^*TP53*^^WT/Null^ and (E) non-cancerous HEK293/IMR-90 cell lines after 24 h treatment with vehicle or DRx-098D-R. (F) Cell viability of HCT116^*TP53*^^Null^ treated for 24 h with vehicle, DRx-097A-R, or idasanutlin. (G) HCT116^*TP53*^^Null^ co-stained for MDM2 (647 nm) and MDMX (488 nm), counterstained with DAPI (nuclei) (scale bar, 10 μm). ns, not significant; ∗*p* < 0.05; ∗∗*p* < 0.01; ∗∗∗*p* < 0.001; ∗∗∗∗*p* < 0.0001.

It is well documented that targeting the p53 pathway in *TP53* MT cancer is ineffective and represents a persistent barrier to existing MDM2/MDMX-p53 targeted therapeutics due to their inability to exploit MDM2/MDMX’s p53-independent mechanisms. Given the growing recognition of p53-independent, pro-oncogenic MDM2/MDMX signaling, we sought to investigate the anti-proliferative consequence of disrupting MDM2 dimerization against a panel of *TP53* WT and MT human cancer cell lines from a broad range of cancer lineages (solid and hematological, Figures 3A and 3B). Interestingly, DRx-098D-R significantly inhibited the relative cell viability of all cell lines with similar growth IC_50_ values, excluding PANC1 (*TP53* R273W). Importantly, negative control peptide DRx-097A-R had no effect on cell viability (Figures 3A and 3B). Corresponding assessment of endogenous MDM2 and MDMX protein expression in each of the solid cancer cell lines highlighted that PANC1 expressed significantly lower levels of combined MDM2 and MDMX (Figure 3C). These findings may suggest a dependence on combined MDM2/MDMX expression for DRx-098D-R activity.

To determine if DRx-098D-R anti-proliferative activity translated in the context of *TP53* null cancer, *TP53* WT and null HCT116 cancer cell lines were utilized. DRx-098D-R significantly inhibited relative cell viability of both *TP53* WT and null HCT116 with similar potency (Figure 3D). Cytotoxicity was not observed in HEK293 and IMR-90 cells (statistically compared to their respective vehicle control, Figure 3E). As demonstrated previously with nutlin-3a, idasanutlin had no anti-proliferative activity against HCT116 *TP53* null cells (Figure 3F).^14^^,^^15^ Consistent with Figure 3C, MDM2 and MDMX protein expression were clearly observed in HCT116 *TP53* null cells (Figure 3G).

## Discussion

MDM2 and MDMX possess pro-oncogenic functions beyond p53 regulation, with overexpression observed in *TP53* null cancers to promote cancer cell progression.^1^^,^^16^^,^^17^^,^^18^ Consequently, MDM2/MDMX depletion and E3 ligase inhibition has been reported to induce cell-cycle arrest and apoptosis in these contexts.^10^^,^^19^^,^^20^ Existing MDM2/MDMX inhibitors predominantly target the interaction with p53 and thus fail to modulate MDM2/MDMX p53-independent functions. “Next generation” MDM2 PROTACs offer improvement upon these inhibitors.^21^^,^^22^ However, as their MDM2-targeting “warheads” are predominantly derived from the same p53-MDM2/MDMX small molecules, they lack the ability to bind pro-oncogenic splice variants of MDM2 that do not contain the N-terminal p53-binding domain. Over 40 MDM2 splice variants have identified, most of which lack the p53-binding domain while retaining their transformative ability.^23^ A key example being MDM2-B, the most frequently expressed transcript, that comprises only of the C-terminal RING domain and is well validated as promoting tumorigenesis in a p53 null setting.^16^^,^^24^ Consequently, there remains a major unmet need for truly broad-reaching MDM2/MDMX inhibitors for the treatment of both MDM2/MDMX-dependent *TP53* WT and MT cancers.

Here, we demonstrate a more comprehensive mechanistic approach to exploiting MDM2/MDMX pro-oncogenic activity through targeting MDM2 homodimer and heterodimer disruption, where anti-cancer activity observed with DRx-098D-R indicates MDM2 dimerization as a potential therapeutic vulnerability in both *TP53* WT and MT cancer. The dimerization interface within the C-terminal RING domain of MDM2 and MDMX, in which DRx-098D-R is designed to engage, is present in all known MDM2/MDMX isoforms. Disrupting MDM2’s dimerization interface therefore offers an unparalleled mechanistic approach to inducing broad MDM2/MDMX neutralization in cancer. Therefore, this proof-of-concept study not only builds upon existing data highlighting this potentially promising therapeutic approach^25^^,^^26^^,^^27^^,^^28^ but also paves the way for future development of a first-in-class MDM2 dimerization disruptor peptide, capable of treating a broad spectrum of MDM2/MDMX-dependent cancers, irrespective of TP53 mutational status. Key focus areas of said development should seek to fully interrogate the therapeutic utility of disrupting MDM2 dimerization in MDM2/MDMX-dependent *TP53* MT cancer, as well as the associated p53-independent mechanisms at play.

## Materials and methods

Please see the supplemental information for the full methods.

## Data availability

All relevant data needed to determine the conclusions stated within the manuscript are available in the main text or the supplemental information. Other raw data/materials used in this study are available upon request to the corresponding author.

## Acknowledgments

We would like to thank Prof. Karen H. Vousden for help and advice on MDM2-MDMX-p53 cellular biology and biochemistry experiments and for gifting plasmids and cell lines. The research was funded by 10.13039/501100000265Medical Research Council (MR/X502807/1) and 10.13039/100016917Scottish Enterprise funding (PS730591C).

## Author contributions

Conceptualization: C.M.B., G.S.B., H.W., and D.T.H. Methodology and investigation: C.M.B., S.F.C., T.A.W., G.L., E.K., Y.Y.S., J.L., A.Z., B.O., W.B., and C.-J.C. Visualization: C.M.B. and S.F.C. Funding acquisition: C.M.B. and G.S.B. Administration: C.M.B., S.F.C., G.S.B., H.W., and D.T.H. Supervision: C.M.B., G.S.B., H.W., and D.T.H. Writing (original): C.M.B. and S.F.C. Writing (revision(s)): C.M.B., S.F.C., G.S.B., H.W., and D.T.H.

## Declaration of interests

D.T.H. is a consultant for Triana Biomedicines. C.M.B. and G.S.B. hold patent rights to relevant published work.

## Supplemental information

Document S1. Supplemental materials and methods

Document S2. Article plus supplemental information
