# Supplementary material for: Targeting MDM2 homodimer and heterodimer disruption with DRx-098D in TP53 wild-type and mutant cancer cells
Source: Mol Ther Oncol. 2025 Aug 6;33(3):201029. doi: 10.1016/j.omton.2025.201029 (PMC12395054; doi:10.1016/j.omton.2025.201029)
Supplement: Document S2. Article plus supplemental information [file mmc2.pdf]

Molecular Therapy
Oncology
Brief report

Targeting MDM2 homodimer and heterodimer disruption with DRx-098D in *TP53* wild-type and mutant cancer cells

Sean F. Cooke,[1,2] Thomas A. Wright,[1] Gillian Lappin,[1] Elka Kyurkchieva,[1] Yuan Yan Sin,[1] Jiayue Ling,[1] Alina Zorn,[1] Bria O'Gorman,[1] William Banyard,[1] Chih-Jung Chang,[1] Helen Wheadon,[2] Danny T. Huang,[3] George S. Baillie,[1] and Connor M. Blair[1]

[1]College of Medical, Veterinary and Life Sciences, University of Glasgow, G12 8QQ Glasgow, UK; [2]Paul O'Gorman Leukaemia Research Centre, University of Glasgow, G11 0YN Glasgow, UK; [3]Cancer Research UK – Scotland Institute, G61 1BD Glasgow, UK

Novel pharmacological strategies capable of inhibiting pro-oncogenic MDM2 beyond its p53-dependent functions represent increasingly attractive therapeutic strategies to treat solid and hematological cancers that are dependent upon MDM2/MDMX, regardless of *TP53* mutational status. Utilizing a novel first-in-class cell-penetrating peptide disruptor of MDM2 homo- and heterodimerization (DRx-098D), we demonstrate the anti-proliferative potential of blocking MDM2 dimerization against a panel of human cancer cell lines that are *TP53* wild type, mutant, or null. DRx-098D elicits its anti-cancer activity via a differentiated mechanism vs. idasanutlin (a phase 3 clinical candidate MDM2-p53 small-molecule inhibitor), inducing significantly superior growth inhibition against *TP53* null HCT116 cells. Our preliminary data highlight, for the first time, the potential therapeutic utility of exploiting both MDM2 homo- and heterodimerization in *TP53* wild-type and mutant cancers with an MDM2-derived disruptor peptide.

INTRODUCTION

Growing recognition of the pro-oncogenic influence, which MDM2 and MDMX have in promoting cancer survival and metastasis, independent of p53, exemplifies the urgent unmet need for novel therapeutic approaches to exploiting MDM2 and MDMX activity.[1–3] Current clinical candidate MDM2-targeting therapeutics (idasanutlin, milademetan, siremadlin, brigimadlin, and sulanemadlin) specifically block MDM2/MDMX's ability to negatively regulate p53 transcriptional activity and promote its degradation via the ubiquitin proteosome system.[4,5] Though this approach has proven successful in *TP53* wild-type (WT) cancer, it does not translate to cancers harboring a *TP53* mutation (MT).[5] In this context, MDM2/MDMX's role in suppressing p53 diminishes. Instead, MDM2/MDMX continue to drive tumorigenesis through a myriad of p53-independent mechanisms, an area of research that remains within its infancy.[1–5]

MDM2 forms homodimers (MDM2:MDM2) and heterodimers (MDM2:MDMX) via its C-terminal RING domain, essential for its enzymatic E3 ligase activity.[6–9] Consequently, pharmacologically targeting the de-stabilization/disruption of C-terminal RING domain dimerization is considered an attractive and underexploited approach to inhibiting MDM2/MDMX, offering a potentially viable therapeutic strategy against MDM2/MDMX-dependent cancers, irrespective of *TP53* mutational status.[5,10]

DRx-098D-R is a novel investigative cell-penetrating MDM2-derived peptide designed to disrupt MDM2:MDM2 and MDM2:MDMX at the dimerization interface. This proof-of-concept study outlines DRx-098D-R's ability to (1) directly bind MDM2 and MDMX, (2) disrupt intracellular MDM2 homo- and heterodimerization, (3) inhibit MDM2 E3 ligase activity, and (4) promote cancer cell death across a broad spectrum of *TP53* WT and MT human cancer cell models. These data re-enforce the therapeutic value of targeting the disruption of pathological protein complexes in disease and precision medicine.[11–13]

RESULTS

DRx-098D-R directly binds MDM2 and MDMX RING-C-terminal truncate proteins

MDM2 forms homodimers and heterodimers with MDMX through its RING-C-terminal domain, and a disruptor peptide, DRx-098D, was designed to target this region (Figure 1A). To confirm target engagement, glutathione S-transferase (GST) fusion MDM2 (428-C) or MDMX(428-C) protein was co-incubated with increasing concentrations (0.05–3 μM) of N-FITC-labeled DRx-098D (DRx-098D-F) or DRx-097A (DRx-097A-F, negative control "knockout" peptide) (Figures 1B and 1C). Unlike DRx-097A-F, DRx-098D-F directly bound to immobilized MDM2 (K_d = 0.774 μM) and MDMX (K_d = 0.294 μM).

Received 27 February 2025; accepted 1 August 2025;
https://doi.org/10.1016/j.omton.2025.201029.

Correspondence: Connor M. Blair, College of Medical, Veterinary and Life Sciences, University of Glasgow, G12 8QQ Glasgow, UK.
E-mail: connor.blair@glasgow.ac.uk

Published by Elsevier Inc. on behalf of The American Society of Gene and Cell Therapy.
This is an open access article under the CC BY license (http://creativecommons.org/licenses/by/4.0/).

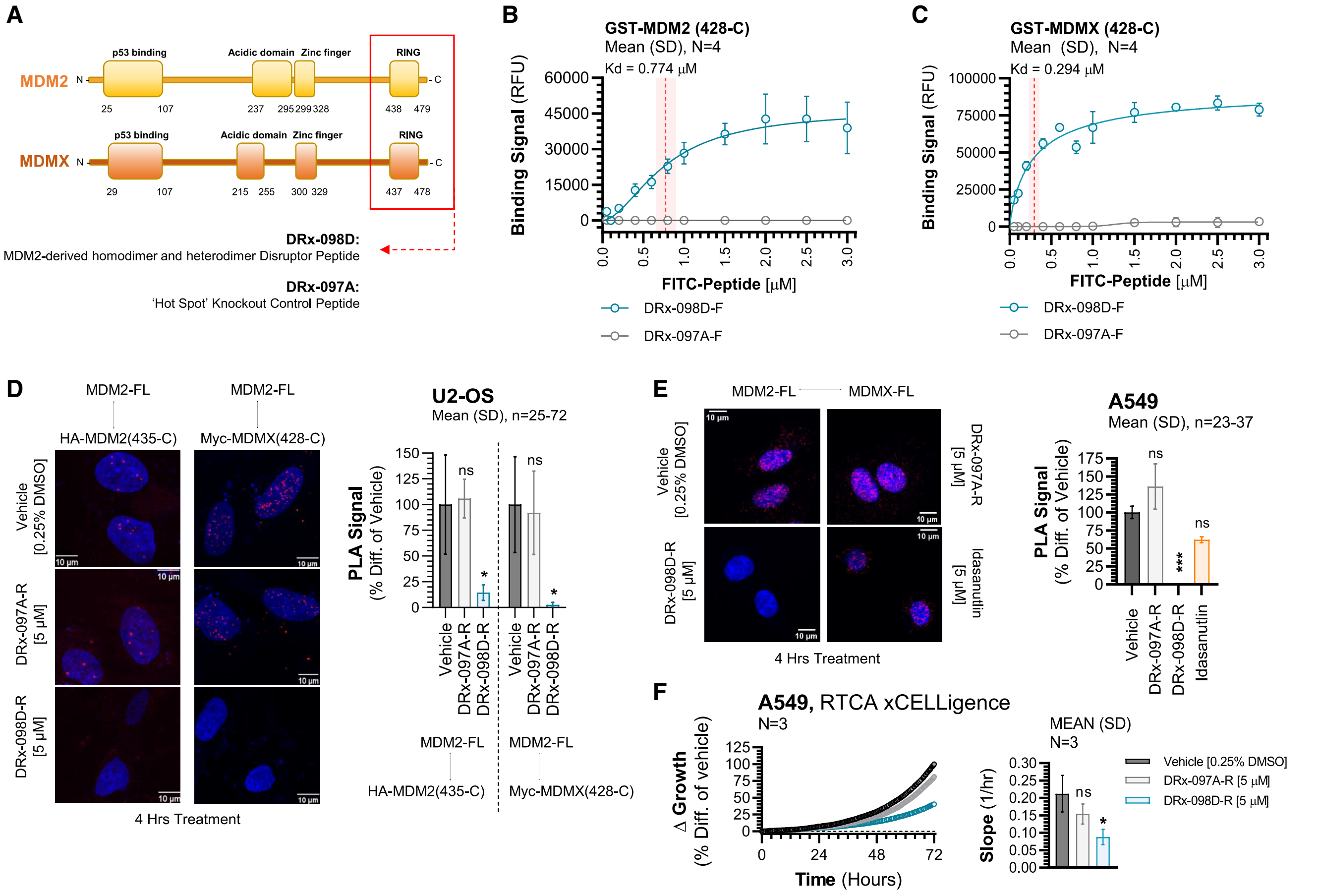

Figure 1. Validation of DRx-098D on-target binding and PPI disruption

(A) MDM2/MDMX structural domains. Red box highlights the MDM2:MDMX RING-C-terminal domain exploited by DRx-098D. Target engagement of (B) GST-MDM2 (428-C) and (C) GST-MDMX (428-C) with FITC-labeled DRx-098D and DRx-097A. (D) Proximity ligation assay (PLA) of full-length (FL) MDM2 and HA-MDM2(435-C) or Myc-MDMX (428-C) in U2-OS, treated with vehicle, DRx-098D-R, or DRx-097A-R. (E) PLA of endogenous FL-MDM2 and MDMX in A549, treated with vehicle, DRx-098D-R, DRx-097A-R, or idasanutlin. (F) A549 real-time cell growth analysis (xCELLigence) following 72 h treatment with vehicle, DRx-098D-R, and DRx-097A-R. ns, not significant; *$p < 0.05$; **$p < 0.01$; ***$p < 0.001$.

DRx-098D-R disrupts MDM2 homodimer and heterodimer formation

To achieve cell permeability, DRx-098D was conjugated to a short-sequence cationic (arginine rich) peptide (DRx-098D-R) and assessed for its ability to block endogenous and exogenous MDM2 homo-/heterodimers. In U2-OS cells (*TP53* WT) overexpressing HA-MDM2(435-C), DRx-098D-R significantly downregulated homodimer formation with full-length (FL) endogenous MDM2 (Figure 1D). This was also observed in U2-OS cells overexpressing Myc-MDMX(428-C), where heterodimerization with FL-MDM2 was significantly inhibited. DRx-097A-R did not affect MDM2 dimer formation. In A549 cells (*TP53* WT), DRx-098D-R significantly attenuated endogenous MDM2 heterodimerization (Figure 1E). Again, this was not observed with DRx-097A-R. Idasanutlin, an MDM2-p53 inhibitor, also did not affect MDM2 heterodimerization, further differentiating DRx-098D-R's MDM2 inhibitory mechanism. Notably, disrupting MDM2 heterodimerization with DRx-098D-R (but not DRx-097A-R) significantly inhibited relative A549 cell growth (Figure 1F).

MDM2 dimer disruption promotes p53 stabilization and upregulates apoptotic pathways

To determine whether disrupting MDM2 dimerization would hinder its E3 ligase activity, DRx-098D-R was assessed in a cell-free *in vitro* MDM2-p53 ubiquitination assay (Figure 2A). DRx-098D-R, but not DRx-097A-R, induced a dose-dependent inhibition of p53 ubiquitination. Thus, DRx-098D-R can inhibit MDM2's E3 ligase activity.

In *TP53* WT cancer, current MDM2/MDMX-p53 inhibitors promote cellular apoptosis as a consequence of upregulated p53 tumor suppressor expression/activity. DRx-098D-R-mediated MDM2 dimer disruption appears consistent with this mechanism, significantly upregulating MDM2, p53, and p21 protein expression (not

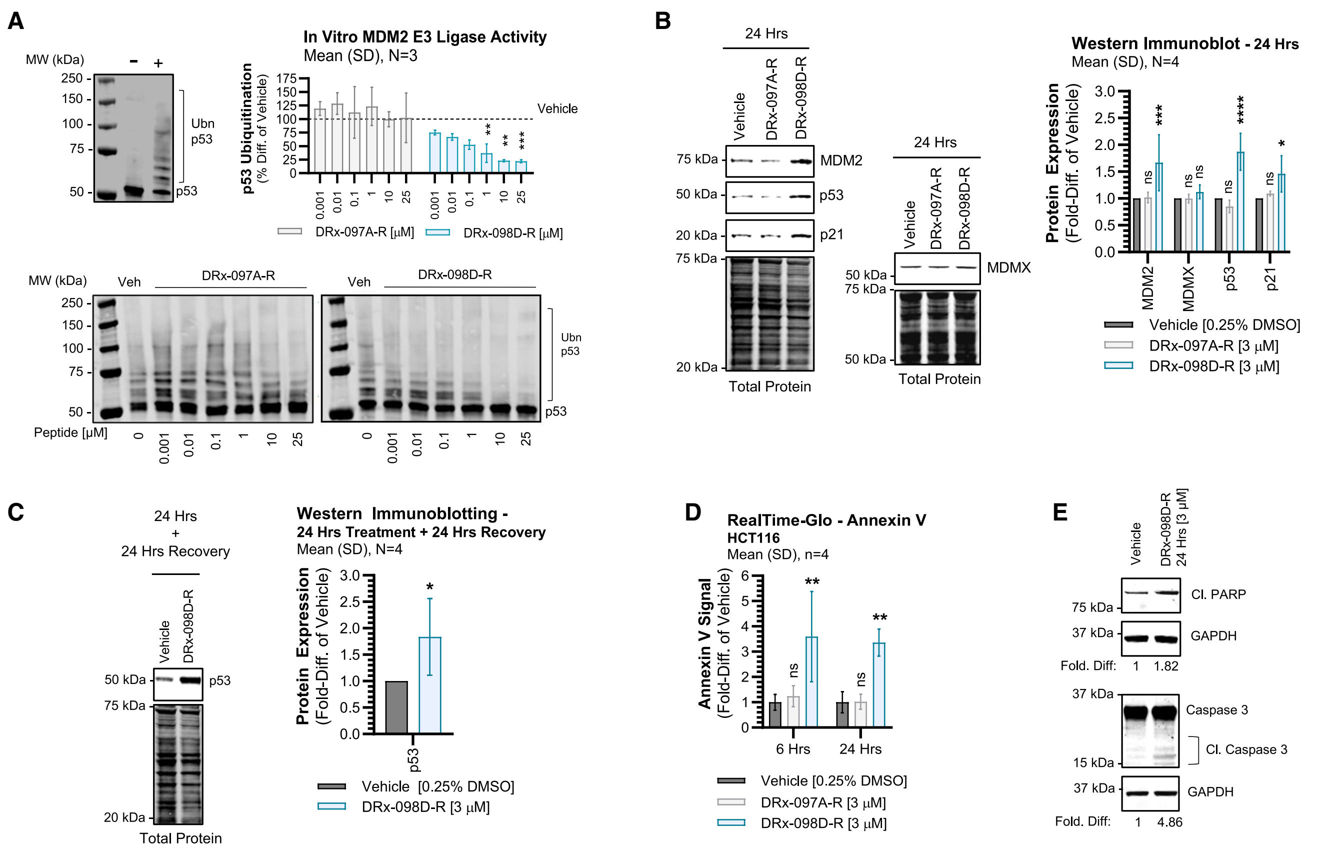

Figure 2. DRx-098D effect on p53 regulation and apoptosis

(A) *In vitro* MDM2 E3 ligase activity assay assessing p53 ubiquitination via western immunoblotting analysis. Increasing concentrations of DRx-098D-R or DRx-097A-R were added to the reaction, and relative MDM2-mediated p53 ubiquitination was measured as a percentage difference of vehicle. Negative (−) indicates no Mg^{2+-}ATP was in reaction mixture (negative control). Positive (+) indicates all reagents, including Mg^{2+-}ATP, were in reaction mixture (positive control) (B) MDM2, MDMX, p53, and p21 protein expression in HCT116$^{TP53\ WT}$ following 24 h treatment with vehicle, DRx-097A-R, and DRx-098D-R or (C) 24 h treatment +24 h wash-out with vehicle and DRx-098D-R (expression normalized to total protein). (D) Annexin V levels in HCT116$^{TP53\ WT}$ measured after 6 and 24 h treatment with vehicle, DRx-097A-R, and DRx-098D-R. (E) Cleaved PARP and cleaved caspase-3 protein levels in HCT116$^{TP53\ WT}$ after 24 h treatment with vehicle and DRx-098D-R. ns, not significant; *$p < 0.05$; **$p < 0.01$; ***$p < 0.001$; ****$p < 0.0001$.

MDMX) (Figure 2B). In addition, DRx-098D-R-induced elevated p53 levels were sustained following a 24 h treatment recovery period (Figure 2C). DRx-098D-R also promoted increased expression of pro-apoptotic markers Annexin V, cleaved PARP, and cleaved caspase-3 (Figures 2D and 2E).

DRx-098D-R is anti-proliferative in *TP53* WT and MT/null cell lines

It is well documented that targeting the p53 pathway in *TP53* MT cancer is ineffective and represents a persistent barrier to existing MDM2/MDMX-p53 targeted therapeutics due to their inability to exploit MDM2/MDMX's p53-independent mechanisms. Given the growing recognition of p53-independent, pro-oncogenic MDM2/MDMX signaling, we sought to investigate the anti-proliferative consequence of disrupting MDM2 dimerization against a panel of *TP53* WT and MT human cancer cell lines from a broad range of cancer lineages (solid and hematological, Figures 3A and 3B). Interestingly, DRx-098D-R significantly inhibited the relative cell viability of all cell lines with similar growth IC_{50} values, excluding PANC1 (*TP53* R273W). Importantly, negative control peptide DRx-097A-R had no effect on cell viability (Figures 3A and 3B). Corresponding assessment of endogenous MDM2 and MDMX protein expression in each of the solid cancer cell lines highlighted that PANC1 expressed significantly lower levels of combined MDM2 and MDMX (Figure 3C). These findings may suggest a dependence on combined MDM2/MDMX expression for DRx-098D-R activity.

To determine if DRx-098D-R anti-proliferative activity translated in the context of *TP53* null cancer, *TP53* WT and null HCT116 cancer cell lines were utilized. DRx-098D-R significantly inhibited relative cell viability of both *TP53* WT and null HCT116 with similar potency (Figure 3D). Cytotoxicity was not observed in HEK293 and IMR-90 cells (statistically compared to their respective vehicle control, Figure 3E). As demonstrated previously with nutlin-3a, idasanutlin

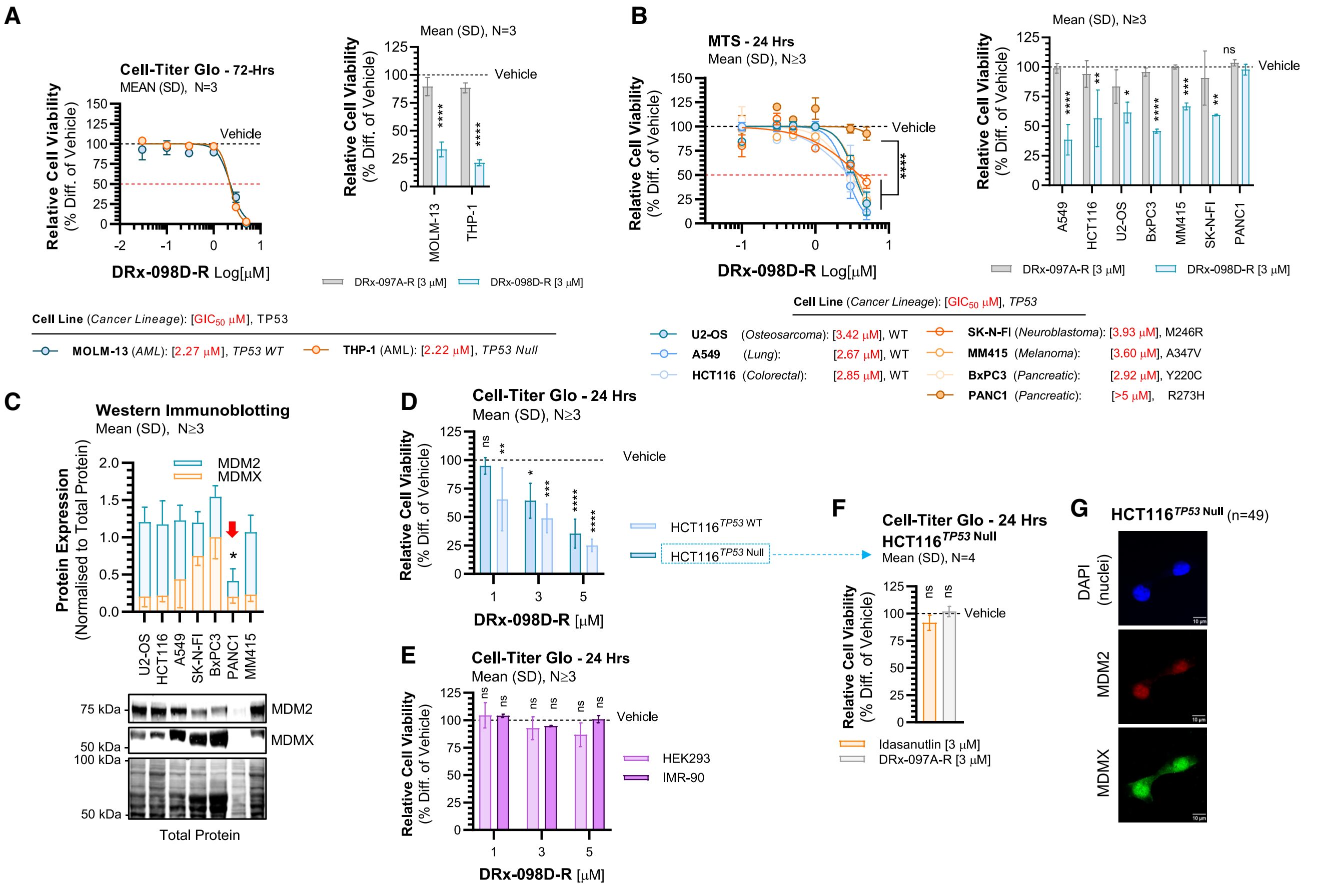

Figure 3. DRx-098D vs. *TP53* WT and MT/null human cancer cell lines

(A) Cell viability of human acute myeloid leukemia (AML) cell lines treated with vehicle, DRx-098D-R, or DRx-097A-R for 72 h. (B) Cell viability of a panel of *TP53* WT and MT human cancer cell lines, treated with vehicle, DRx-098D-R, or DRx-097A-R for 24 h. Relative growth (G) IC_{50} (red) is given below. (C) Combined MDM2 and MDMX protein expression. (D) Cell viability of cancerous HCT116$^{TP53\ WT/Null}$ and (E) non-cancerous HEK293/IMR-90 cell lines after 24 h treatment with vehicle or DRx-098D-R. (F) Cell viability of HCT116$^{TP53\ Null}$ treated for 24 h with vehicle, DRx-097A-R, or idasanutlin. (G) HCT116$^{TP53\ Null}$ co-stained for MDM2 (647 nm) and MDMX (488 nm), counterstained with DAPI (nuclei) (scale bar, 10 μm). ns, not significant; *$p < 0.05$; **$p < 0.01$; ***$p < 0.001$; ****$p < 0.0001$.

had no anti-proliferative activity against HCT116 *TP53* null cells (Figure 3F).[14,15] Consistent with Figure 3C, MDM2 and MDMX protein expression were clearly observed in HCT116 *TP53* null cells (Figure 3G).

DISCUSSION

MDM2 and MDMX possess pro-oncogenic functions beyond p53 regulation, with overexpression observed in *TP53* null cancers to promote cancer cell progression.[1,16–18] Consequently, MDM2/MDMX depletion and E3 ligase inhibition has been reported to induce cell-cycle arrest and apoptosis in these contexts.[10,19,20] Existing MDM2/MDMX inhibitors predominantly target the interaction with p53 and thus fail to modulate MDM2/MDMX p53-independent functions. "Next generation" MDM2 PROTACs offer improvement upon these inhibitors.[21,22] However, as their MDM2-targeting "warheads" are predominantly derived from the same p53-MDM2/MDMX small molecules, they lack the ability to bind pro-oncogenic splice variants of MDM2 that do not contain the N-terminal p53-binding domain. Over 40 MDM2 splice variants have identified, most of which lack the p53-binding domain while retaining their transformative ability.[23] A key example being MDM2-B, the most frequently expressed transcript, that comprises only of the C-terminal RING domain and is well validated as promoting tumorigenesis in a p53 null setting.[16,24] Consequently, there remains a major unmet need for truly broad-reaching MDM2/MDMX inhibitors for the treatment of both MDM2/MDMX-dependent *TP53* WT and MT cancers.

Here, we demonstrate a more comprehensive mechanistic approach to exploiting MDM2/MDMX pro-oncogenic activity through targeting MDM2 homodimer and heterodimer disruption, where anti-cancer activity observed with DRx-098D-R indicates MDM2 dimerization as a potential therapeutic vulnerability in both *TP53* WT and MT cancer. The dimerization interface within the C-terminal RING

domain of MDM2 and MDMX, in which DRx-098D-R is designed to engage, is present in all known MDM2/MDMX isoforms. Disrupting MDM2's dimerization interface therefore offers an unparalleled mechanistic approach to inducing broad MDM2/MDMX neutralization in cancer. Therefore, this proof-of-concept study not only builds upon existing data highlighting this potentially promising therapeutic approach[25–28] but also paves the way for future development of a first-in-class MDM2 dimerization disruptor peptide, capable of treating a broad spectrum of MDM2/MDMX-dependent cancers, irrespective of TP53 mutational status. Key focus areas of said development should seek to fully interrogate the therapeutic utility of disrupting MDM2 dimerization in MDM2/MDMX-dependent *TP53* MT cancer, as well as the associated p53-independent mechanisms at play.

MATERIALS AND METHODS

Please see the supplemental information for the full methods.

DATA AVAILABILITY

All relevant data needed to determine the conclusions stated within the manuscript are available in the main text or the supplemental information. Other raw data/materials used in this study are available upon request to the corresponding author.

ACKNOWLEDGMENTS

We would like to thank Prof. Karen H. Vousden for help and advice on MDM2-MDMX-p53 cellular biology and biochemistry experiments and for gifting plasmids and cell lines. The research was funded by Medical Research Council (MR/X502807/1) and Scottish Enterprise funding (PS730591C).

AUTHOR CONTRIBUTIONS

Conceptualization: C.M.B., G.S.B., H.W., and D.T.H. Methodology and investigation: C.M.B., S.F.C., T.A.W., G.L., E.K., Y.Y.S., J.L., A.Z., B.O., W.B., and C.-J.C. Visualization: C.M.B. and S.F.C. Funding acquisition: C.M.B. and G.S.B. Administration: C.M.B., S.F.C., G.S.B., H.W., and D.T.H. Supervision: C.M.B., G.S.B., H.W., and D.T.H. Writing (original): C.M.B. and S.F.C. Writing (revision(s)): C.M.B., S.F.C., G.S.B., H.W., and D.T.H.

DECLARATION OF INTERESTS

D.T.H. is a consultant for Triana Biomedicines. C.M.B. and G.S.B. hold patent rights to relevant published work.

SUPPLEMENTAL INFORMATION

Supplemental information can be found online at https://doi.org/10.1016/j.omton.2025.201029.

REFERENCES

1. Jones, S.N., Hancock, A.R., Vogel, H., Donehower, L.A., and Bradley, A. (1998). Overexpression of Mdm2 in mice reveals a p53-independent role for Mdm2 in tumorigenesis. Proc. Natl. Acad. Sci. USA *95*, 15608–15612.
2. Klein, A.M., de Queiroz, R.M., Venkatesh, D., and Prives, C. (2021). The roles and regulation of MDM2 and MDMX: it is not just about p53. Genes Dev. *35*, 575–601.
3. Kung, C.P., and Weber, J.D. (2022). It's Getting Complicated-A Fresh Look at p53-MDM2-ARF Triangle in Tumorigenesis and Cancer Therapy. Front. Cell Dev. Biol. *10*, 818744.
4. Mullard, A. (2020). p53 programmes plough on. Nat. Rev. Drug Discov. *19*, 497–500.
5. Haronikova, L., Bonczek, O., Zatloukalova, P., Kokas-Zavadil, F., Kucerikova, M., Coates, P.J., Fahraeus, R., and Vojtesek, B. (2021). Resistance mechanisms to inhibitors of p53-MDM2 interactions in cancer therapy: can we overcome them? Cell. Mol. Biol. Lett. *26*, 53.
6. Linares, L.K., Hengstermann, A., Ciechanover, A., Müller, S., and Scheffner, M. (2003). HdmX stimulates Hdm2-mediated ubiquitination and degradation of p53. Proc. Natl. Acad. Sci. USA *100*, 12009–12014.
7. Kawai, H., Lopez-Pajares, V., Kim, M.M., Wiederschain, D., and Yuan, Z.M. (2007). RING Domain–Mediated Interaction Is a Requirement for MDM2's E3 Ligase Activity. Cancer Res. *67*, 6026–6030.
8. Nomura, K., Klejnot, M., Kowalczyk, D., Hock, A.K., Sibbet, G.J., Vousden, K.H., and Huang, D.T. (2017). Structural analysis of MDM2 RING separates degradation from regulation of p53 transcription activity. Nat. Struct. Mol. Biol. *24*, 578–587.
9. Egorova, O., Lau, H.H., McGraphery, K., and Sheng, Y. (2020). Mdm2 and MdmX RING Domains Play Distinct Roles in the Regulation of p53 Responses: A Comparative Study of Mdm2 and MdmX RING Domains in U2OS Cells. Int. J. Mol. Sci. *21*, 1309.
10. Klein, A.M., Biderman, L., Tong, D., Alaghebandan, B., Plumber, S.A., Mueller, H.S., van Vlimmeren, A., Katz, C., and Prives, C. (2021). MDM2, MDMX, and p73 regulate cell-cycle progression in the absence of wild-type p53. Proc. Natl. Acad. Sci. USA *118*, e2102420118.
11. Carvajal, L.A., Neriah, D.B., Senecal, A., Benard, L., Thiruthuvanathan, V., Yatsenko, T., Narayanagari, S.R., Wheat, J.C., Todorova, T.I., Mitchell, K., et al. (2018). Dual inhibition of MDMX and MDM2 as a therapeutic strategy in leukemia. Sci. Transl. Med. *10*, eaao3003.
12. Sorolla, A., Wang, E., Golden, E., Duffy, C., Henriques, S.T., Redfern, A.D., and Blancafort, P. (2020). Precision medicine by designer interference peptides: applications in oncology and molecular therapeutics. Oncogene *39*, 1167–1184.
13. Darvishi, E., Ghamsari, L., Leong, S.F., Ramirez, R., Koester, M., Gallagher, E., Yu, M., Mason, J.M., Merutka, G., Kappel, B.J., and Rotolo, J.A. (2022). Anticancer Activity of ST101, A Novel Antagonist of CCAAT/Enhancer Binding Protein β. Mol. Cancer Ther. *21*, 1632–1644.
14. Lakoma, A., Barbieri, E., Agarwal, S., Jackson, J., Chen, Z., Kim, Y., McVay, M., Shohet, J.M., and Kim, E.S. (2015). The MDM2 small-molecule inhibitor RG7388 leads to potent tumor inhibition in p53 wild-type neuroblastoma. Cell Death Discov. *1*, 15026.
15. Humpton, T.J., Hock, A.K., Maddocks, O.D.K., and Vousden, K.H. (2018). p53-mediated adaptation to serine starvation is retained by a common tumour-derived mutant. Cancer Metab. *6*, 18.
16. Steinman, H.A., Burstein, E., Lengner, C., Gosselin, J., Pihan, G., Duckett, C.S., and Jones, S.N. (2004). An Alternative Splice Form of Mdm2 Induces p53-independent Cell Growth and Tumorigenesis. J. Biol. Chem. *279*, 4877–4886.
17. Xiong, S., Pant, V., Zhang, Y., Aryal, N.K., You, M.J., Kusewitt, D., and Lozano, G. (2017). The p53 inhibitor Mdm4 cooperates with multiple genetic lesions in tumourigenesis. J. Pathol. *241*, 501–510.
18. Dembla, V., Somaiah, N., Barata, P., Hess, K., Fu, S., Janku, F., Karp, D.D., Naing, A., Piha-Paul, S.A., Subbiah, V., et al. (2018). Prevalence of MDM2 amplification and coalterations in 523 advanced cancer patients in the MD Anderson phase 1 clinic. Oncotarget *9*, 33232–33243.
19. Feeley, K.P., Adams, C.M., Mitra, R., and Eischen, C.M. (2017). Mdm2 is required for survival and growth of p53-deficient cancer cells. Cancer Res. *77*, 3823–3833.
20. Uldrijan, S., Pannekoek, W.J., and Vousden, K.H. (2007). An essential function of the extreme C-terminus of MDM2 can be provided by MDMX. EMBO J. *26*, 102–112.
21. Wang, W., Albadari, N., Du, Y., Fowler, J.F., Sang, H.T., Xian, W., McKeon, F., Li, W., Zhou, J., and Zhang, R. (2024). MDM2 Inhibitos for Cancer Theraoy: The Past, Present and Future. Pharmacol. Rev. *76*, 414–453.
22. Li, H., Cai, X., Yang, X., and Zhang, X. (2024). An overview of PROTACs targeting MDM2 as a novel approach for cancer therapy. Eur. J. Med. Chem. *272*, 116506.
23. Bartel, F., Taubert, H., and Harris, L.C. (2002). Alternative and aberrant splicing of MDM2 mRNA in human cancer. Cancer Cell *2*, 9–15.
24. Jones, S.N., Hancock, A.R., Vogel, H., Donehower, L.A., and Bradley, A. (1998). Overexpression of Mdm2 in mice reveals a p53-independent role for Mdm2 in tumorigenesis. Proc. Natl. Acad. Sci. USA *95*, 15608–15612.

25. Pellegrino, M., Mancini, F., Lucà, R., Coletti, A., Giacchè, N., Manni, I., Arisi, I., Florenzano, F., Teveroni, E., Buttarelli, M., et al. (2015). Targeting the MDM2/MDM4 interaction interface as a promising approach for p53 reactivation therapy. Cancer Res. *75*, 4560–4572.

26. Wu, W., Xu, C., Ling, X., Fan, C., Buckley, B.P., Chernov, M.V., Ellis, L., Li, F., Muñoz, I.G., and Wang, X. (2015). Targeting RING domains of Mdm2-MdmX E3 complex activates apoptotic arm of the p53 pathway in leukemia/lymphoma cells. Cell Death Dis. *6*, e2035.

27. Merlino, F., Pecoraro, A., Longobardi, G., Donati, G., Di Leva, F.S., Brignola, C., Piccarducci, R., Daniele, S., Martini, C., Marinelli, L., et al. (2024). Development and Nanoparticle-Mediated Delivery of Novel MDM2/MDM4 Heterodimer Peptide Inhibitors to Enhance 5-Fluorouracil Nucleolar Stress in Colorectal Cancer Cells. J. Med. Chem. *67*, 1812–1824.

28. Ballarotto, M., Bianconi, E., Valentini, S., Temperini, A., Moretti, F., and Macchiarulo, A. (2024). Rational design, synthesis, and biophysical characterization of a peptidic MDM2-MDM4 interaction inhibitor. Bioorg. Med. Chem. *113*, 117937.

OMTON, Volume *33*

Supplemental information

Targeting MDM2 homodimer and heterodimer disruption with DRx-098D in *TP53* wild-type and mutant cancer cells

Sean F. Cooke, Thomas A. Wright, Gillian Lappin, Elka Kyurkchieva, Yuan Yan Sin, Jiayue Ling, Alina Zorn, Bria O'Gorman, William Banyard, Chih-Jung Chang, Helen Wheadon, Danny T. Huang, George S. Baillie, and Connor M. Blair

Supplemental Information

Materials and Methods:

Antibodies and Chemicals. Primary antibodies include p53 (Santa Cruz, sc-126, 1:1000 – WB), MDM2 (Cell Signaling, 86934, 1:500 – WB, 1:200 – ICC/PLA), MDM2 (Abcam, ab216895, 1:50, PLA), MDMX (Sigma, HP-048821, 1:500 – WB, 1:250 – ICC/PLA), Myc (Cell Signaling, 2276, 1:200 – ICC/PLA), HA (Cell signaling, 2367, 1:200 – ICC/PLA), p21 (Santa Cruz, sc-6246, 1:1000 – WB), Caspase 3 (Cell Signaling, 9662, 1:1000 – WB), Cleaved PARP (Cell Signaling, 5625, 1:1000 – WB), GAPDH (Millipore, MAB374, 1:3000 – WB). Secondary antibodies include donkey anti-mouse 800nm (LI-COR Biosciences, 926-32212, 1:10,000 – WB), donkey anti-rabbit 800nm (LI-COR Biosciences, 926-32213, 1:10,000 – WB), goat anti-mouse 680nm (LI-COR Biosciences, 926-68070, 1:10,000 – WB), Alexa Fluor donkey anti-mouse 647nm (Thermo, A68072), Alexa Fluor donkey anti-rabbit 488nm (Thermo, A-48269). For western immunoblotting (WB), antibodies were diluted in Intercept T20 TBS antibody diluent (LI-COR). For ICC, antibodies were diluted in 5% donkey serum, 0.5% BSA in PBS. For PLA, antibodies were diluted in Duolink® antibody diluent (Merck). Stock concentrations of Idasanutlin (R&D Systems), DRx-098D (Cambridge Research Biochemicals) and DRx-097A (Cambridge Research Biochemicals) were diluted in 100% DMSO to [10 mM]. Compounds were further diluted to $\leqslant$1% DMSO in PBS or media in all assays. Unless otherwise state, drug treatments were carried out in low serum conditions (2% FBS). Idasanutlin is a clinical candidate small molecule inhibitor of the MDM2 – p53 protein-protein interaction. DRx-098D is short linear peptide (<2 kDa) that has been designed to selectively target the disruption of MDM2:MDM2 homodimer and MDM2:MDMX heterodimer formation through mimicking the binding interface. Unlike MDMX-derived peptides reported in [25, 27-28], DRx-098D was derived from the dimerisation interface of MDM2. DRx-098D-F is fluorescently labelled with an N-terminal FITC. DRx-098D-R possess enhanced cell permeability through linkage of a short-sequence (arginine rich) cationic peptide at the N-terminus of DRx-098D. DRx-097A represents the respective DRx-098D negative control peptide, where the known binding 'hot spot' residues have been substituted out to significantly negate target engagement.

Due to an on-going patent application submitted on behalf of the University of Glasgow associated with DRx-098D-R (and related intellectual property), the authors (of which include inventors on said patent) have been advised to limit structural disclosures of MDM2 disruptor peptides, de-risking/enabling patentability.

Target Engagement. Incubated overnight at 4°C, 50 ng of GST-tagged MDM2(428-C) or MDMX(428-C) truncate proteins (purified as described previously [8]) were immobilised to glutathione coated wells of a pre-blocked, clear-bottom, black 96-well plate (15340, Thermo). Increasing concentrations of FITC-labelled peptide [0.05 – 3 μM] were then added to respective wells and incubated for 2 Hrs at room temperature. Wells were washed 3 times in 1X TBS-T following each incubation step to remove excess protein/peptide. Proteins and peptides were incubated in the same binding buffer (200 mM NaCl, 50 mM Tris, 5% glycerol, 5 mM DTT, 0.01% tween-20, 5 mg/mL BSA, pH 7.5). A Tristar 5 multimode microplate reader (Berthold Technologies) was utilised to measure FITC-peptide binding to MDM2 and MDMX protein. Non-linear regression analysis was performed to measure binding affinities (Kd) using GraphPad Prism 8.0 software.

C-terminal RING MDM2 protein sequence (aa428-491):
SSLPLNAIEPCVICQGRPKNGCIVHGKTGHLMACFTCAKKLKKRNKPCPVCRQPIQMIVLTYFP

C-terminal RING MDMX protein sequence (aa428-490):
DCQNLLKPCSLCEKRPRDGNIIHGRTGHLVTCFHCARRLKKAGASCPICKKEIQLVIKVFIA

In Vitro Ubiquitination. A cell-free, *in vitro*, human MDM2 ubiquitin ligase – p53 substrate kit (R&D Systems, K-200B) was used, as per manufacturer's instructions, to assess relative MDM2 E3 ligase activity. MDM2 E3 ligase activity was assessed in the absence and presence of DRx-098D-R [0.001 – 25 μM], DRx-097A-R [0.001 – 25 μM] or Vehicle (DMSO). MDM2 E3 ligase activity was determined through p53 ubiquitination, observed/quantified utilising SDS-PAGE western immunoblotting (see protocol below). Densitometry was carried out (Image J) on non-ubiquitinated p53 (i.e., single protein band at approx. 50 kDa) and the respective smear/additional bands above (i.e., ubiquitinated p53). Relative p53 ubiquitination was normalised to respective non-ubiquitinated p53 and represented as a % difference of vehicle control (100%).

Cell Culture. PANC1 (ATCC – CRL-1469), U2-OS (ATCC – HTB-96), HCT116 TP53 wild-type, HCT116 TP53 null, SK-N-FI (ATCC – CRL-2142), IMR-90 (ATCC – CCL-186) and HEK293 (ATCC – CRL-1573) cell lines were cultured in complete DMEM. BxPC3 (ATCC – CRL-1687), MM415 (Sigma - 10092319) and A549 (ATCC – CCL-185) were cultured in complete RPMI. All media were made complete following supplementation with 2 mM L-Glutamine, 10% FBS and 100 U/mL Pen-Strep. SK-N-FI was also supplemented with 1% MEM non-essential amino acids. All cell lines were cultured in a humidified environment with 5% CO_2 at 37°C. U2-OS, HCT116 TP53 wild-type and HCT116 *TP53* null were gifted from Prof Karen H. Vousden's research group (Francis Crick Institute, London, UK).

Immunocytochemistry. HCT116 *TP53* null cells were seeded at 0.5 x 10^5 cells per well of a 12-well plate containing a sterilised 0.13-0.17mm glass coverslip in complete DMEM and incubated overnight. Cells were fixed in 4% paraformaldehyde (Sigma) for 15 minutes at room temperature, then permeabilised with 0.1% triton X100 (Sigma) for 4 minutes at room temperature. Cells were then blocked in 10% donkey serum, 1% BSA in PBS for 1 Hr at room temperature. Following blocking, cells were co-incubated in MDM2 (mouse) and MDMX (rabbit) primary antibodies overnight at 4°C. Subsequently, secondary Alexa Fluor antibodies were co-incubated for a further 1 Hr at room temperature. Cells were washed in PBS three times between each step. Following final wash, coverslips were mounted onto glass slides with Prolong Gold Antifade Mountant with DAPI (Thermo, P36941), stored in dark overnight at room temperature, and imaged using a Zeiss (LSM880) confocal microscope the following day (63X objective).

Proximity Ligation Assay. U2-OS cells were seeded at 1.5 x 10^5 cells per well of a 6-well plate containing sterilised glass coverslips (0.13-0.17mm) in complete (10% FBS) media and incubated overnight. U2-OS cells were then transiently transfected with 2.5 µg (pcDNA3.1+) HA-MDM2(435-C) or Myc-MDMX(428-C) plasmid DNA (Prof. Karen H. Vousden, Francis Crick Institute, London, UK (20)) for 48 Hrs using Lipofectamine P3000 reagent as per manufacturer's instructions (Invitrogen). U2-OS cell media was then replaced with low serum (2% FBS) media containing appropriate concentration of vehicle (0.25% DMSO), DRx-098D-R [5 µM] or DRx-097A-R [5 µM] for 4 Hrs. In contrast, A549 cells were seeded at 0.5 x 10^5 cells per well of a 12-well plate, and cultured/treated in high serum (10% FBS) media. Following treatments, cells were fixed, permeabilised and washed as per immunocytochemistry protocol. Blocking and subsequent in situ detection of MDM2 dimerisation was then carried as per Duolink® proximity ligation manufacturer's instructions (Merck, DUO92008). Cells were counterstained with DAPI and imaged as outlined in immunocytochemistry protocol.

C-terminal RING MDM2 sequence (aa435-491):
IEPCVICQGRPKNGCIVHGKTGHLMACFTCAKKLKKRNKPCPVCRQPIQMIVLTYFP

C-terminal RING MDMX sequence (aa428-490):
DCQNLLKPCSLCEKRPRDGNIIHGRTGHLVTCFHCARRLKKAGASCPICKKEIQLVIKVFIA

RTCA xCELLigence. Real-time cellular analysis (RTCA) of A549 cells was measured utilising the label-free cellular growth xCELLigence platform (Roche Applied Science, Agilent Technologies), where-by cellular impedance was leveraged as an indirect indicator of relative cell growth (i.e., cell index; CI). A549 cells were seeded at 1 x 10^4 cells per well of a 96-well E-plate and allowed to adhere/grow overnight in complete (10% FBS) media. Cells were then treated with vehicle (0.25% DMSO), DRx-097A-R [5 µM] or DRx-098D-R [5 µM] for 72 Hrs, measuring cellular growth (CI) every 15 minutes. CI was normalised to 1 at treatment timepoint, and then again to 0% relative growth at time point 0. Relative growth (Δ growth) of treated A549 cells was assessed as a % difference of vehicle after 72 Hrs post-treatment (i.e., 100% relative cell growth at experiment endpoint).

Cell Viability. For MOLM-13, THP-1 (Fig. 3A), HCT116 *TP53* wild-type, HCT116 *TP53* null (Fig. 3D), HEK293, and IMR-90 (Fig. 3E) cells, relative cell viability was assessed via CellTiter Glo 2.0 Cell Viability Assay (Promega: G9241), as per manufacturer's instructions. For U2-OS, A549, HCT116, BxPC3, MM415, SK-N-FI, PANC1 cells (Fig. 3B), relative cell viability was assessed via CellTiter 96® Aqueous One Solution Cell Proliferation Assay (MTS, Promega: G3581), as per manufacturer's instructions. All adherent cell lines were seeded at 5 x 10^3 per well of a 96-well plate (MTS assay = clear plate, Cell Titer-Glo assay = clear bottom white plate) in low serum (2% FBS) media and cultured overnight. In the case of MOLM-13 and THP-1, cells were seeded at 2.5 x 10^4 cells per well. Adherent cell lines were then treated for 24 Hrs in Vehicle (0.25% DMSO) or appropriate concentration of DRx-097A-R, DRx-098D-R, or Idasanutlin. 0.05% triton X100 was utilised as a cytotoxic 'kill' control (0%). In the case of MOLM-13 and THP-1, cells were treated for 72 Hrs. Relative cell viability was represented as a % difference of vehicle (100%). Cell viability was measured (MTS assay = absorbance – 490nm, Cell Titer-Glo assay = luminescence) using a Tristar 5 multimode microplate reader (Berthold Technologies).

Western Immunoblotting. Following harvesting of protein lysates utilising appropriate lysis buffer (25 mM Tris, 150 mM NaCl, 0.1 mM EDTA, 1% NP-40, 5% glycerol, pH 7.5, protease inhibitor, phosphatase inhibitor), samples were diluted in 5X SDS gel loading buffer (10% SDS, 300 mM Tris-HCl, 0.05% bromophenol blue, 10% β-mercaptoethanol) and boiled for 5 minutes at 90°C. Protein resolution was carried out via SDS-PAGE (30 µg per lane, 4-12% 10-well Bis-Tris gels (NuPAGE)), transferred onto nitrocellulose membranes (GE Healthcare), and blocked in Intercept TBS blocking buffer (LI-COR). Blocked membranes were incubated in appropriate primary antibody overnight at 4°C, followed by 1 Hr incubation in appropriate secondary antibody at room temperature. An Odyssey CLx imaging system (LI-COR) was utilised to visualise immunoreactive bands, following which densitometry of immunoreactive bands was carried (Image J). All proteins were normalised to respective Revert™ 700nm Total Protein Stain (LI-COR, 926-11011) or GAPDH.

Annexin V Assay. HCT116 *TP53* wild-type cells were seeded at 5 x 10^3 per well of a white, clear-bottom 96-well plate and incubated overnight. Cells were then treated with DRx-097A-R [3 µM], DRx-098D-R [3 µM] or Vehicle

(0.25% DMSO) for 6 Hrs or 24 Hrs. Following treatments, Annexin V levels were measured utilising the RealTime-Glo^{TM} Annexin V Apoptosis luminescence-based assay (Promega, JA1000) as per manufacturer's instructions. Luminescent Annexin V levels were measured via a Tristar 5 multimode microplate reader (Berthold Technologies). HCT116 cells were incubated and treated in low serum (2% FBS) conditions.

Statistical Analysis. All data were analysed via a one or two-way ANOVA (Dunnett's or Tukey's multiple comparison). Where data is represented as Mean ± SD from ≥3 replicates, significance was determined by a p value < 0.05. GraphPad Prism 8.0 software was utilised to statistically analysis all data.
